# Relationships between nurse managers’ work activities, nurses’ job satisfaction, patient satisfaction, and medication errors at the unit level: a correlational study

**DOI:** 10.1186/s12913-021-06288-5

**Published:** 2021-04-01

**Authors:** Anu Nurmeksela, Santtu Mikkonen, Juha Kinnunen, Tarja Kvist

**Affiliations:** 1 Faculty of Health Sciences, Department of Nursing Science, University Teacher, University of Eastern Finland, P.O. Box 1627, 70211 Kuopio, Finland; 2 Department of Applied Physics and Department of Environmental and Biological Sciences, University of Eastern Finland, P.O. Box 1627, 70211 Kuopio, Finland; 3 Central Finland Central Hospital, Keskussairaalantie 19, 40620 Jyväskylä, Finland; 4 Department of Nursing Science, Faculty of Health Sciences, University of Eastern Finland, Kuopio Campus, P.O. Box 1627, 70211 Kuopio, Finland

**Keywords:** Job satisfaction, Medication errors, Nurses, Nurse manager, Patient satisfaction, Hospital

## Abstract

**Background:**

Nurse managers play a critical role in enhancing nursing and patient outcomes. The work of nurse managers, who can be described as middle-managers at health care organizations, is complex and changes on a daily basis. Only a few studies have clarified how nurse managers divide their time across various work activities. This study aimed to describe the relationships between nurse managers’ work activities, nurses’ job satisfaction, patient satisfaction, and medication errors at the hospital unit level.

**Methods:**

A cross-sectional and correlational study design was used. The data were collected from nurse managers (*n* = 29), nursing staff (*n* = 306), and patients (*n* = 651) from 28 units across three Finnish acute care hospitals between April and November 2017. In addition, data concerning medication errors (*n* = 468) over one calendar year (2017) were acquired from the hospitals’ incident reporting register. Analysis of covariance (ANCOVA) was used to estimate relationships between data from subareas of Nurse Managers’ Work Content Questionnaire, Kuopio University Hospital Job Satisfaction Scale, and Revised Humane Caring Scale, along with medication error reports. A significance level of 95% was applied when estimating the covariances between variables. Unstandardized regression coefficients (B) were used to explain the relationships between variables.

**Results:**

Multiple relationships between nurse managers’ work activities, nurses’ job satisfaction, patient satisfaction, and medication errors were identified. Nurse managers’ work activities had both positive and negative relationships on the other studied variables. The *Requiring factors of work* (*p <* .001) subarea of nurses’ job satisfaction, total patient satisfaction (*p <* .001), and medication errors (*p <* .001) were identified as the variables most significantly affected by other factors.

**Conclusions:**

The findings suggest that nurse managers should focus on improving nursing practices by managing and organizing nurses’ work in a way that makes their employees feel supported, motivated and secure. Furthermore, nurse managers should adopt a leadership style that emphasizes safe and patient-centered care. The results also suggest that the administration of today’s health care organizations should actively evaluate nurse managers’ share of work activities to ensure that their daily work is in line with the organizational goals.

**Supplementary Information:**

The online version contains supplementary material available at 10.1186/s12913-021-06288-5.

## Background

Nurse managers’ work has become increasingly demanding in the current health care environment [1]. Nurse managers largely influence nurses’ job satisfaction [2, 3] and patient safety [4, 5], while motivated and engaged staff improve patient satisfaction [6, 7]. Overall, nurse managers’ work and behavior affect nursing outcomes in complex ways.

Only a limited number of studies have investigated how nurse managers divide their time across professional work activities [8–13], with a few studies focusing on how frequently nurse managers perform certain work activities [14–18]. Nevertheless, previous literature has shown that nurse managers have various responsibilities and duties, ranging from staff recruitment and daily management to strategic planning and financial management [13]. In recent years, nurse managers have become more involved in administrative work while their share of clinical work has diminished [11, 17, 18]. Recent studies have reported that nurse managers’ daily work often consists of organizing, work scheduling and resource management [13, 19, 20]. Nurse managers can impact the quality of care [21] by ensuring that their unit has sufficient staff and actively participates in the recruitment of competent staff [22–25].

Today, communication and collaboration represent a considerable part of nurse managers’ work [10, 11, 23, 26–30]. Cadmus and Wisniewska (2013) discovered that nurse managers most frequently perform rounds in their unit, guide staff on clinical matters, and have short meetings, or “huddles”, with staff on a daily basis [15]. Sveinsdottir et al. (2018) found that nurse managers’ common daily activities also included “other domains”, such as telephone calls, participating in planned meetings, and responding to e-mail. Furthermore, Chen et al. (2020) found that nurse managers frequently participate in information management on a daily basis. However, increased workloads among nurse managers have reduced the time they can share with nurses [23, 31–33]. This presents a challenge, as nurse managers need to be visible and approachable, as well as give regular feedback to their staff [26, 34, 35]. As nurse managers are also tasked with promoting work protection [20, 32], work safety activities for staff [33, 36, 37], and a healthy work environment [28], the finding from Chen et al. (2020) that nursing managers’ daily work also includes patient management and overseeing nursing quality could be considered as completely logical. Although it is recognized that patient safety culture is influenced by hospital-level predictors, e.g., hospital size and staff education levels [38, 39], nurse managers nevertheless have an important role in patient safety at the unit level (Cummings et al., 2018).

Nevertheless, there is scarce research about how the activities that nurse managers perform are related to nursing outcomes. Instead, most of the available research covers how a nurse manager’s leadership style and work behavior influence nursing outcomes. Multiple studies have identified a positive link between the relational leadership style and nurses’ job satisfaction [3, 6, 40], while other research has linked this leadership approach with patient satisfaction [2, 3, 41]. In addition, it is challenging for nurse managers to lead quality improvement in the complex everday environment of a health care organization [42]. Recent studies have shown that leadership, managerial support and nurse-manager trust reduce medication errors and increase both patient safety culture and the quality of care [5, 6, 43].

In summary, the current literature on nurse managers’ leadership suggests that there are some relationships between hospital-level predictors and nursing outcomes, but the dynamics underlying these relationships may be highly complex. However, research regarding the relationships between nurse managers’ work activities and nursing outcomes is not available. Due to the limited knoweldge base, this study aimed to describe the relationships between nurse managers’ work activities, nurses’ job satisfaction, patient satisfaction, and medication errors at the hospital unit level. The research question underlying the present study was: What are the relationships between nurse managers’ work activities, nurses’ job satisfaction, patient satisfaction, and medication errors?

## Methods

### Study design and participants

This study applied a cross-sectional and correlational design. The research applied convenience sampling. A total of 104 nurse managers from three Finnish acute care hospitals were invited to participate in the survey, with 61 answering the questionnaire. All of the nurses (*N* = 3225) and 50 patients (*N* = 3050) from each unit in which the participating nurse managers worked were invited to take part in the study. The inclusion criterion for respondents was that they were either a nurse manager or a nurse at an inpatient ward or outpatient department. More specifically, to be eligible, a nurse had to be a registered nurse, midwife, practical nurse or mental health nurse. The exclusion criterion for nurse managers and nurses was working in an operating room, intensive care unit or paedatric unit. The inclusion criteria for patients were an adult patient who was being discharged from an inpatient ward or outpatient department and the ability to answer the questionnaire by him/herself. The exclusion criteria for patients were children patients and patients in the intensive care unit or operating room. The inclusion criterion for pooled units was that at least one nurse manager, three or more nurses, and three or more patients from the same unit had answered the survey. Register data describing the medication errors which had occurred over 1 year (2017) were acquired from the hospitals’ incident reporting register. Data regarding nurse managers, nurses, patients and medication errors were pooled by every unit. After all of the inclusion and exclusion criteria had been considered, a total of 29 nurse managers (one unit was represented by two managers), 306 nurses, and 651 patients across 28 units participated in the study. Furthermore, the study covered 498 incident reports of medication errors.

The first author visited each hospital and presented the study design plan at a nurse directors’ and managers’ meeting. Data were collected between April and November 2017 from nurse managers and nurses by e-mail and from patients by paper questionnaire. The questionnaires for nurse managers and nurses were sent to a contact person at each hospital, who then forwarded the email with the questionnaire link to nurse managers and nurses at the hospital. The paper questionnares for patients were distrubuted to each unit, and nurses were informed that they should give each patient the questionnaire (including a return envelope) when they are being discharged. A patient safety coordinator from each hospital delivered anonymous registered data of medication errors by e-mail or mail. All of the hospitals were public hospitals that offer specialized medical care. The included hospitals had between 390 to 440 beds and 2396 to 3748 employees. In addition, the hospitals had between 1285 and 1928 nursing staff [44].

### Measurements

Data concerning the demographic characteristics of nurse managers, nurses and patients were collected. However, only information about a nurse manager’s hospital, number of subordinates, and age were reported in this study. Nursing staff were described in terms of type of employment, working hours, type of contract and work experience, while patients were described in terms of hospital, gender, age and reason for hospital admission (Table 1).

**Table 1:** Characteristics of nursing staff (*n* = 306) and patients (*n* = 651), described as number (n) and percentage (%)

Nursing staff	n	%
Hospital		
1	98	32.0
2	121	39.5
3	87	28.4
Gender		
female	291	95.1
male	15	4.9
Age (years)		
< 30	37	12.1
30–39	79	25.8
40–49	81	26.5
50–59	88	28.8
60–69	21	6.9
Type of employment		
Permanent	254	83.0
Temporary	52	17.0
Working Hours		
Rotational; three-shift work	187	61.1
Full day	119	38.9
Type of contract		
Full-time employment	258	84.3
Short-term employment	48	15.7
Work experience (years)		
< 10	80	26.1
10–19	106	34.6
≥ 20	120	39.2
**Patients**		
Hospital		
1	151	23.2
2	364	55.9
3	136	20.9
Gender		
female	388	60.0
male	259	40.0
Age (years)		
< 30	78	12.5
30–39	53	8.5
40–49	50	8.0
50–59	95	15.3
60–69	173	27.8
≥ 70	173	27.8
Hospital admission of patients		
Planned	421	58.1
Emergency	224	30.96

A total of three different measures (Table 2), along with register data of medication errors, were used in this study. Furthermore, hospital [1–3] and number of nurses managed by each nurse manager were variables in this study. Nurse Managers’ Work Content Questionnaire (NMWCQ) was used to collect data related to how often nurse managers performed various work activities [18]. Data collection was performed by electronic questionnaire. The NMWCQ was developed in 2016 to identify the content of nurse managers’ work and which tasks they spend the most amount of time on. The questionnaire includes 87 items across 13 subscales, more specifically: *Recruitment* (5 items); *Organizing* (7 items); *Work well-being* (5 items); *Work atmosphere* (3 items); *Communication* (5 items); *Clinical nursing* (9 items); *Development of the unit* (12 items); *Personnel development* (8 items); *Development of nursing* (4 items); *Financial management* (7 items); *Planning and evaluation of activities* (6 items); *Collaboration* (10 items); and *Development with collaborating partners* (6 items) (Additional file 1). The scale employs a six-point ordinal scale (1 = daily; 2 = weekly; 3 = monthly; 4 = 2–4 times a year; 5 = annual; and 6 = never). The development and preliminary results of the questionnaire were reported in an earlier study; as such, the data used in this study represent secondary data. Previous research reported Cronbach’s alpha values between 0.554–0.890 for the NMWCQ [18], while in this study the Cronbach’s alpha values ranged between 0,478–0,916 (Table 2).

**Table 2:** Nurse managers’ work activities (*n* = 29), nurses’ job satisfaction (*n* = 306) and patient satisfaction (*n* = 651) presented according to subscale, and described using mean score, standard deviation (SD), and Cronbach’s alpha

Scale (number of items)	n	Mean	SD	α	scale
**Nurse managers’ work activities (NMWCQ)**					
Recruitment (5)	29	3.2875	.88377	0.842	
Organizing (7)	29	4.6224	.66350	0.767	(1–6):
Work well-being (5)	29	3.4214	.47559	0.738	6 = daily
Work atmosphere (3)	29	3.6429	.77475	0.776	5 = weekly
Communication (5)	29	3.8000	.59129	0.478	4 = monthly
Clinical nursing (9)	29	2.7450	.99992	0.817	3 = 2–4 times a year
Development of the unit (12)	29	4.0418	.78712	0.916	2 = annual
Personnel development (8)	29	3.4281	.68550	0.769	1 = never
Development of nursing (4)	29	3.7232	.80029	0.840	
Financial management (7)	29	3.3010	.78375	0.782	
Planning and evaluation of activities (6)	29	3.4464	.62370	0.779	
Collaboration (10)	29	3.9066	.75205	0.835	
Development with collaborating partners (6)	29	3.8869	.49908	0.656	
**Job satisfaction (KUHJSS)**					
Leadership (7)	305	7.275	1.998	0.950	0–10:
Requiring factors of work (8)	303	6.340	1.648	0.843	0 = not satisfied at all
Motivating factors of the work (5)	301	8.461	1.154	0.816	10 = completely satisfied
Working welfare (4)	304	7.992	1.296	0.723	
Participation in decision-making (4)	303	6.492	1.889	0.815	
Sense of community (4)	304	7.473	1.639	0.811	
Working environment (4)	304	7.178	1.432	0.766	
**Patient satisfaction (RHCS)**					
Professional practice (17)	650	9.155	1.098	0.970	0–10:
Information and participation in own care (11)	650	8.813	1.387	0.946	0 = not satisfied at all
Cognition of physical needs (4)	590	8.741	1.803	0.846	10 = completely satisfied
Human resources (3)	642	8.512	1.775	0.881	
Pain and apprehension (4)	621	8.356	1.917	0.786	
Interdisciplinary collaboration (3)	645	9.153	1.162	0.916	
Outcomes variables (4)	644	8.929	1.479	0.894	

*Abbreviations*: *n* number of participants, *SD* standard deviation, *α* Cronbach’s Alpha

Kuopio University Hospital Job Satisfaction Scale (KUHJSS) was used to measure nurses’ job satisfaction. The data were collected via an electronic questionnaire [45]. The KUHJSS includes 15 background questions and seven subscales, namely, *Leadership* (7 items), *Requiring factors of work* (8 items), *Motivating factors of the work* (6 items), *Working welfare* (4 items), *Participation in decision-making* (4 items), *Sense of community* (4 items), and *Working environment* (4 items) (Additional file 2). The subscales include a total of 37 continuous scale questions, which respondents score from 0 to 10, i.e., totally disagree (0) – totally agree [10]. Exploratory factor analysis was used to test the internal consistency of the instrument [45], while instrument validity and reliability were evaluated in several other studies. Cronbach’s alpha values between 0.64–0.92 have previously been calculated for the KUHJSS [45, 46], while in the present study Cronbach’s alpha values ranged between 0.723–0.95 (Table 2).

The Revised Humane Caring Scale (RHCS) was used to measure patient satisfaction.) [47, 48]. The data were collected through a paper questionnaire. This instrument includes seven background questions and seven subscales, namely, *Professional practice* (17 items), *Information and participation in own care* (11 items), *Cognition of physical needs* (4 items), *Human resources* (3 items), *Pain and apprehension* (4 items), *Interdisciplinary collaboration* (3 items), and *Outcomes variables* (4 items) (Additional file 3). These seven subscales include a total of 46 items, which respondents grade from 0 to 10, i.e., totally disagree (0) – totally agree [10]. Cronbach’s alpha values between 0.775–0.946 have been reported for the RHCS [47, 48]. In this study, the Cronbach’s alpha values were between 0.786–0.970 .

Data concerning medication errors during the year 2017 were acquired from the hospitals’ incident reporting register (HaiPro). HaiPro is a national, web-based patient safety reporting system launched in 2007. Today, over 200 Finnish health- and social-care organizations report medication errors in HaiPro [49].

### Ethical considerations

Ethics committee approval was obtained from the University of Eastern Finland. Approval was also requested, and received, from each of the three hospitals prior to data collection. Furthermore, the General Data Protection Regulation was followed throughout the research [50]. Nurse managers, nurses and patients were informed of the voluntary nature of the study and that data would be anonymously analyzed. In addition, the registered data describing medication errors were anonymous.

### Data analysis

Frequencies, percentages and means were used to describe the demographic variables.

Mean scores were calculated for the NMWCQ, KUHJSS and RHCS subscales while frequencies were used to describe medication errors. In addition, Cronbach’s alpha values were calculated for the subscales of the NMWCQ, KUHJSS and RHCS to describe the internal consistency of questionnaires. Missing data were not replaced for any of the scales used. During data analysis, a Spearman’s correlation matrix was first used to identify correlations between nurse managers’ performed work activities, nurses’ job satisfaction, and patient satisfaction. This analysis assesses the monotonic relationship – instead of the linear relationship - between two variables and also allows ordinal variables to be included in the analysis [51]. Subscales with correlation coefficients ≥0.3 were included in the covariance analysis.

ANCOVA is a statistical approach that is able to include both categorical and continuous predictors in a single model [51]. This was necessary for our data as the studied predictors contain both types of variables. ANCOVA was used to evaluate the relationships between the NMWCQ, KUHJSS, and RHCS subscales, along with hospital, the number of nurses per nurse manager and medication errors in one unit [51]. The KUHJSS and RHCS subscales, along with medication errors, were applied as dependent variables and ANCOVA was used to test how these variables were affected by the subscales identified during the correlation analysis, as well as hospital and the number of nurses per nurse manager. The NMWCQ, KUHJSS, and RHCS subscales, along with medication errors, were included as predictor variables for each other, i.e., NMWCQ subscales were included as covariates for the KUHJSS and RHCS subscales and medication errors. An individual predictor was included in the ANCOVA model if the significance level *p* < 0.1. Furthermore, hospital size and the number of nurses per nurse manager were used as fixed factors in the ANCOVA. Unstandardized regression coefficients (B) were used to explain the relationship between predictor and dependent variable. Furthermore, the original scale of the NMWCQ (1 = daily, 2 = weekly, 3 = monthly, 4 = 2–4 times a year, 5 = annual, 6 = never) was reversed to improve the interpretation of results, i.e. the reversed scale was: 6 = daily; 5 = weekly; 4 = monthly; 3 = 2–4 times a year; 2 = annual; and 1 = never. The data analyses were performed in SPSS for Windows (version 25.0, IBM Corporation, Armonk, NY).

## Results

### Demographic characteristics

The results represent 28 units, including responses from 29 nurse managers, 306 nurses, and 651 patients (Table 1). Each unit was generally represented by one nurse manager, with the exception of one unit which was represented by two nurse managers. The responding nurse managers, nurses and patients had average ages of 51, 46, and 57 years, respectively. Nurse managers were – on average - in charge of 35 nurses (range: 14–60).

### Means scores of NMWCQ, KUHJSS and RHCS subscales

The mean score for nurse managers’ work activities was 3.61 (on a scale of 1–6), with *Clinical working* being the least frequently performed activity (2.75) and *Organizing* being the most frequently performed activity (4.62). Nurses’ total job satisfaction was 7.36 (on a scale of 0–10), with the *Requiring factors of work* and *Motivating factors of the work* subscales receiving the lowest (6.34) and highest (8.46) mean scores, respectively. The mean score for total patient satisfaction was 8.74 (on a scale of 0–10), with the *Human resources* and *Profes*s*ional practice* subscales showing the lowest (8.51) and highest (9.16) scores, respectively (Table 2).

### Models of job satisfaction, patient satisfaction and medication errors

The ANCOVA yielded six different models of nurses’ job satisfaction (Table 3), eight different models of patient satisfaction (Table 4), and one model of medication errors (Table 5). These models are presented below, along with descriptions of how the variables included in each are related to nurse managers’ work activities.

**Table 3:** The relationships of hospital, number of nurses, nurse managers’ work activities (NMWCQ), patient satisfaction (RHCS) and medication errors on nurses’ job satisfaction (KUHJSS) subareas at the unit (*n* = 28) level

The model of job satisfaction (KUHJSS)		B	*p*
Requiring factors of work	Development of nursing (NMWCQ) Cognition of physical needs (RHCS) Outcomes variables (RHCS)	−.623 −.547 .779	*<* .001***
Working environment	Hospitals Hospital 1 Hospital 2 Hospital 3 Number of nurses < 40 > 40 Communication (NMWCQ)	.932 .201 0^a^ −.410 0^a^ −.457	.002**
Leadership	Number of nurses < 40 > 40 Work well-being (NMWCQ) Outcomes variables (RHCS) Medication errors	.654 0^a^ −.413 .966 .022	.047*
Working welfare	Cognition of physical needs (RHCS)	−.239	.025*
Motivating factors of the work	Communication (NMWCQ)	−.306	.050*
Total job satisfaction	Communication (NMWCQ) Outcomes variables (RHCS)	−.301 .403	.044*

Significance: * = *p* < 0.05; ** = *p* < 0.005; *** = *p* < 0.001

*Abbreviations*: *B* Unstandardized coefficients, *NMWCQ* Nurse Managers’ Work Content Questionnaire, *KUHJSS* Kuopio University Hospital Job Satisfaction Scale, *RHCS* Revised Humane Caring Scale

**Table 4:** The relationships between nurse managers’ work activities (NMWCQ), nurses’ job satisfaction (KUHJSS), medication errors and subareas of patient satisfaction (RHCS) at the unit (*n* = 28) level

The model of patient satisfaction (RHCS)		B	*p*
Outcomes variables	Leadership (KUHJSS) Medication errors	.132 −.011	.002**
Interdisciplinary collaboration	Work well-being (NMWCQ) Medication errors	-.171 -.005	.002**
Cognition of physical needs	Development of nursing (NMWCQ) Requiring factors of work (KUHJSS)	-.782 -.543	.003**
Professional practice	Organizing (NMWCQ) Clinical nursing (NMWCQ) Leadership (KUHJSS)	-.124 -.178 -.114	.004**
Pain and apprehension	Communication (NMWCQ) Development of nursing (NMWCQ) Working welfare (KUHJSS) Medication errors	.324 -.327 -.420 -.011	.005**
Information and participation in own care	Organizing (NMWCQ) Medication errors	-.201 -.011	.007**
Human resources	Financial management (NMWCQ) Medication errors	-.273 -.014	.028**
Total patient satisfaction	Work well-being (NMWCQ) Working welfare (KUHJSS) Medication errors	-.217 -.356 -.006	<.001***

Significance: * = *p* < 0.05; ** = *p* < 0.005; *** = *p* < 0.001

*Abbreviations*: *B* Unstandardized coefficients, *NMWCQ* Nurse Managers’ Work Content Questionnaire, *KUHJSS* Kuopio University Hospital Job Satisfaction Scale, *RHCS* Revised Humane Caring Scale

**Table 5:** The relationships of hospital, nurse managers’ work activities (NMWCQ), and patient satisfaction (RHCS) on medication errors at the unit (*n* = 28) level

The model of medication errors		B	*p*
Medication errors	Hospitals Hospital 1 Hospital 2 Hospital 3 Planning and evaluation of activities (NMWCQ) Outcomes variables (RHCS)	9.643 15.058 0^a^ 11.346 −15.816	< .001***

Significance: * = *p* < 0.05; ** = *p* < 0.005; *** = *p* < 0.001

*Abbreviations*: *B* Unstandardized coefficients, *NMWCQ* Nurse Managers’ Work Content Questionnaire, *KUHJSS* Kuopio University Hospital Job Satisfaction Scale, *RHCS* Revised Humane Caring Scale

### Job satisfaction

The results showed that six subareas of nurses’ job satisfaction were related with nurse managers’ work, patient satisfaction and medication errors (Table 3). The most significant effects were found for the *Requiring factors of work* subscale (*p* < .001). For example, high ratings for both a nurse manager’s *Development of nursing* duties and patient assessments of *Cognition of physical needs* were negatively related with this component of nurses’ job satisfaction. The results revealed that nurses’ assessments of general factors of their work were rather poor even though nurse managers were frequently involved in staff orientation and solving patient complaints. Furthermore, patient satisfaction with their physical care was associated with poor ratings of work conditions (e.g. enough staff, satisfaction of working hours) among staff. However, patient views of outcomes were positively associated with nurses’ satisfaction with *Requiring factors of work* (Table 3).

There were inter-hospital differences in terms of nurses’ perceptions of *Working environment* (*p* = .002) (e.g. appropriate work facilities, work unit is safe and secure). Accordingly, nurses from hospital 1 scored this factor of job satisfaction higher than nurses from hospital 2, while nurses from hospital 3 gave this factor the lowest score. A small number of nurses (*n* < 40) per nurse manager was negatively related to nurses’ perceptions of the *Working environment*. In other words, nurses working in small units were less satisfied with their working environment than nurses working in larger units. Furthermore, increased commitment towards *Communication* among nurse managers was negatively related with nurses’ experiences of *Working environment* at the unit level (Table 3).

However, a small number of nurses per nurse manager (n < 40) was positively related with nurses’ perceptions of *Leadership* (*p* = .047). Hence, nurses in small units were more satisfied with their managers’ leadership behavior than nurses working in larger units. In addition, patient ratings of *Outcomes variables* and the number of medication errors were both found to be positively associated with the *Leadership* aspect of nurses’ job satisfaction. This means that patient satisfaction with treatment and outcomes translated to favorable assessment of leadership among nurses even if the unit had high medication errors rates. In contrast, high scores for nurse manager’s *Work well-being* duties were negatively related with nurses’ perceptions of *Leadership*. Both employee sick leaves and early support conversations are included in well-being duties (Table 3).

An increase in patient perceptions of *Cognition of physical needs* slightly decreased nurses’ *Working welfare* (*p* = .025). Accordingly, nurses who worked in an unit where patients needed more physical care evaluated their personal welfare poorly. Furthermore, increased commitment to *Communication* among nurse managers was negatively associated with nurses’ ratings of *Motivating factors of the work* (*p* = .050), as well as nurses’ total job satisfaction (*p* = .044). The amount of time which nurse managers spent in meetings and counsels was negatively related to nurses’ motivation and overall work satisfaction. Patient ratings of *Outcomes variables* was positively correlated with total job satisfaction among nurses (Table 3).

### Patient satisfaction

The analysis showed that eight subareas of patient satisfaction were related with nurse managers’ work activities, nurses’ job satisfaction and medication errors (Table 3). Positive nurse assessments of a nurse manager’s leadership were positively related to the *Outcomes variables* aspect of patient satisfaction (*p* < .002). This means that patients were more satisfied with their care outcomes when nurses were satisfied with their managers’ leadership behavior. In contrast, a high relative number of *Medication errors* in a unit was negatively related with the patient *Outcomes variables* subscale (Table 4).

The frequency at which nurse managers performed *Work well-being* duties and the number of medication errors were both found to decrease patient perceptions of *Interdisciplinary collaboration* (*p* = .002). Nurse managers’ work well-being duties include both promoting health at the workplace and supportive activities for staff. On the other hand, nurse managers’ nursing development duties involve the orientation and training of staff in addition to handling patient complaints. The frequency at which nurse managers participated in *Development of nursing* duties and nurses’ ratings of *Requiring factors of work* were both negatively related to patient perceptions of *Cognition of physical needs* (*p* = .003). This could explain the patients’ views of physical caring. It should be noted that nurses’ asessesments of good work conditions, for example, the sufficiency of employees, may not reflect patients’ experiences. Three factors decreased patient satisfaction with *Professionalism practice* (*p* = .004), namely, a nurse manager’s commitment to *Organizing* and *Clinical nursing* and nurses’ perceptions of *Leadership*, i.e., units in which nurse managers frequently participated in *Organizing* and *Clinical nursing*, and in which nurses were confident with the managers’ leadership, showed lower patient satisfaction relative to other units (Table 4).

Increased commitment to *Communication* among nurse managers was found to improve patient satisfaction with *Pain and apprehension* (*p* = .005). On the other hand, this component of patient satisfaction decreased with the frequency at which nurse managers participate in *Development of nursing* duties, nurses’ perceptions of *Working welfare* and the number of medication errors. Accordingly, an increase in patient complaints and medication errors increased the time that nurse managers spend investigating problems (i.e., *Development of nursing*). Moreover, we identified a seemingly paradoxical inverse relationship between nurses’ work welfare and patient satisfaction with *Pain and apprehension* (Table 4).

Furthermore, the frequency at which nurse managers participated in *Organizing* duties and the number of medication errors were negatively related to patient assessments of *Information and participation in own care* (*p* = .007). In addition, an increase in either a nurse manager’s commitment to *Financial management* or the number of medication errors diminished patient satisfaction with *Human resources* (*p* = .028) (Table 4). Daily organizing is largely focused on scheduling, which is also related to financial resources. In addition, poorly organized work could increase the amount of medication errors at a unit. Therefore, it is logical that these aspects would influence patients’ perceptions of how much time nurses have to guide and inform patients, as well as the extent to which patients are involved in their own care.

An increased focus on *Work well-being* among nurse managers, higher nurse ratings of *Working welfare*, and a greater number of medication errors were all found to decrease total patient satisfaction (*p* = .001) (Table 4). Thus, although a nurse manager’s decision to allot more time to daily supportive duties may improve nurses’ assessments of their work welfare, this decision may also increase medication errors, and therefore, decrease patient satisfaction.

### Medication errors

A total of 468 medication errors occurred across the 28 units during the one-year study period, which translates to an annual average of 17 medication errors per unit (range: 0–75). The results revealed that medication errors at the unit level were related with nurse managers’ work activities, patient satisfaction and the hospital as an organizational factor. However, only two of the tested variables were shown to significantly affect medication errors (*p* < .001). The analysis revealed inter-hospital differences in medication error prevalence, with hospital 2 showing the highest prevalence, as well as significantly more medication errors than hospital 3. Furthermore, the frequency at which nurse managers participated in *Planning and evaluation of activities* (e.g., process improvements) was found to be linked with an increase in medication errors. In contrast, patients’ opinions of *Outcomes variables* were negatively related with medication errors. Consequently, units in which patients were satisfied with the outcomes of care also showed a lower number of medication errors rates than units in which patients were less satisfied with care (Table 5).

To summarize, the performed analyses revealed several relationships between nurse managers’ work activities, nurses’ job satisfaction, patient satisfaction, and medication errors. Nurse managers’ work activities had both positive and negative effects on the studied variables. The *Requiring factors of work* (*p <* .001) aspect of nurses’ job satisfaction, total patient satisfaction (*p <* .001), and medication errors (*p <* .001) were found to be the studied variables that were most significantly affected by other factors.

## Discussion

The participating nurse managers had an average age of 51 years, which is similar to the average age of Finnish nurse managers [52]. The subarea of *Organizing* was found to be the activity most frequently performed by nurse managers. This is in line with previous research, as organizing has been described as an essential part of nurse managers daily duties [11, 13, 20]. The participating nurses had average age of 46 years, which is close to the mean age of nurses in Finland, i.e., 45 years [53, 54]. The participating nurses were most satisfied with the motivating factors of work, and least satisfied with requiring factors. This is consistent with what has been presented in previous studies of job satisfaction among Finnish nurses [46]. The participating patients were generally highly satisfied with the care they received, as has been the case in previous studies [47, 48]. Furthermore, the studied units were found to vary greatly in terms of the number of medication errors. Previous research has also reported that the number of medication errors can vary within a hospital, i.e., between different units [55, 56].

### Job satisfaction

Concerning nurses’ job satisfaction, *Requiring factors of work* was negatively related to the nurse managers’ focus on *Development of nursing* and to patient satisfaction regarding *Cognition of physical needs*, while this aspect of job satisfaction was positively linked to patient views of *Outcomes variables.* A potential explanation is that a nurse manager’s decision to allocate resources to nursing processes, along with the education and orientation of staff, would reduce the resources for bedside nursing, and therefore, may influence nurse staffing. According to several studies, scheduling and organizing are part of nurse managers’ daily work responsibilities [13, 19, 20].

Furthermore, patient satisfaction with *Outcomes variables* was found to be positively related to nurses’ job satisfaction in terms of both *Requiring factors of work* and total job satisfaction. Recent research by De Simone et al. (2018) and Zaghini et al. (2020) provides support for these findings, i.e., both of these studies reported correlations between patient satisfaction and nurses’ job satisfaction [57, 58]. Nurses are motivated to provide high-quality care [48]; as such, it is logical that patient satisfaction with the outcomes of care will improve nurses’ job satisfaction.

When rating the *Working environment* aspect of job satisfaction, nurses evaluate whether they work in facilities that are safe and secure. Fang et al. (2018) found that over one-third of nurses thought that they work with unsafe equipment and did not feel adequately supported, while nearly half of nurses felt unsafe in the workplace. However, additional research found that nurses believe that nurse managers are able to change the work systems and equipment to promote nurse safety [59]. In addition, Agnew et al. (2014) found that a nurse manager’s behavior regarding the monitoring (e.g. auditing) and recognizing (e.g. rewarding) of safety issues influences the compliance of staff. Another study reported that the hospital and number of nurses influence both nurses’ perceptions of the work environment and/or nurse managers’ leadership abilities. Consequently, nurses from units with less staff were more satisfied with their managers’ leadership behavior than nurses from units with more staff. On the other hand, units with less nurses were characterized by lower ratings of the work environment in comparison to units with larger pools of nursing staff. The nursing practice environment has been found to impact staff perceptions of staffing and resource adequacy. However, staffing is not the sole reason for dissatisfaction among nurses. For example, dissatisfaction can also be the result of poor leadership and management, lack of lifelong learning opportunities, poor nurse empowerment, an insecure work environment, and strained nurse-physician relationships [60]. In addition, other organizational factors - such as environment or culture, organizational support, and staffing adequacy – can contribute to nurses’ job satisfaction [40, 61].

The frequency at which nurse managers perform *Communication* tasks was found to be negatively related to nurses’ total job satisfaction, along with the following aspects of nurses’ job satisfaction: *Motivating factors of the work; Working environment*; and *Leadership*. The subarea of *Communication* includes preparing for and participating in meetings, managing unit meetings, and conversations with personnel. These findings were similar to the results reported by Kirchhoff & Karlsson (2019), more specifically, nurse managers who frequently engage in meetings with management, such as networking with other managers and involvement in management-level projects, were less visible in the organizational unit [31]. Several studies have reported that nurse managers need to be visible, accessible, and provide regular feedback to their staff [26, 34, 35]. This could be the reason why nurses were less motivated and satisfied when their managers were highly focused on communication tasks. An alternative explanation is that a large proportion of nurses felt that multiple staff meetings were unnecessary and unmeaningful. These results suggest that nurses managers should focus on their communication skills, e.g. discussing difficult questions, listening to different opinions, delivering construtive feedback, and disseminating up-to-date information, rather than the time they spend on communication tasks [27, 62].

### Patient satisfaction

The performed analyses revealed that total patient satisfaction was significantly related to nurse managers’ *Work well-being,* nurses’ *Working welfare* and medication errors. This means that patients are satisfied when nurse managers treat staff members equally, are interested in staff well-being, provide staff feedback with the aim of developing work, and are interested in work results and outcomes [19]. Hence, nurse managers influence patient satisfaction in various ways.

Nurses’ satisfaction with *Leadership* demonstrated a positive relationship with patients’ *Outcomes variables*, which describes the goals of treatment and satisfaction with outcomes and care, while the number of medication errors had negative influence on this aspect of patient satisfaction. For example, an increase in nurses’ perceptions of their nurse managers’ leadership behavior could be expected to improve patient outcomes. Several previous studies have also confirmed that nurse managers’ leadership is related to nurses’ job satisfaction [40, 63, 64]. Furthermore, other studies have linked nurses’ job satisfaction with patient outcomes [65] and patient satisfaction [6, 61].

An interesting finding of this study was that the frequency at which nurse managers performed numerous tasks had a negative impact on different components of patient satisfaction. For example, a nurse manager’s decision to dedicate more time to *Organizing*, *Work well-being*, *Work atmosphere*, *Financial management*, *Clinical nursing* or *Development of nursing care* was found to decrease at least one subscale of patient satisfaction. However, it should be noted that most of these observed decreases were rather slight. In contrast, a nurse manager’s focus on *Communication* improved patient evaluations of *Pain and apprehension*. It is also important to note that the frequency at which a nurse manager performs a certain task does not necessarily denote an improvement in the quality of work. For example, several recent studies have emphasized that nurse managers are overwhelmed by their workloads. According to Steege et al. (2017), fatigue among nurse managers decreases the quality of their work, and can impact decision-making [66]. On the other hand, research by Labrague et al. (2018) suggests that – in some cases - more control over a job, along with a higher extent of responsibility, lead to less occupational stress. For these reasons, it is important to review and evaluate how nurse managers’ work activities are scheduled, and concentrate on developing collaboration with colleagues and supervisors.

### Medication errors

Several of the tested variables were significantly related to the incidence of medication errors. These included the frequency at which nurse managers performed certain tasks, patient satisfaction, and the studied hospital, each of which affected the incidence of medication errors at the unit level. There were large inter-hospital differences, as hospitals 1 and 2 had nearly 10 and 15 times more medication errors, respectively, than hospital 3. Another important finding was that the frequency at which nurse managers participated in *Planning and evaluating activities* significantly increased the amount of medication errors at a unit. Nurse managers are responsible for the fluency of nursing processess and ensuring that all staff members understand the organizational goals. Consequently, they connect the clinical environment with the organizational culture. Accordingly, units with strong patient safety culture are characterized by organizational learning, continuous improvement, nonpunitive responses to errors, as well as feedback and open communication, and therefore, have a lower incidence of adverse events than units that do not perform as strongly across these safety culture aspects. Furthermore, these environments include an atmosphere in which employees feel safe to report medication errors, discuss them, and learn from previous mistakes [3, 67]. Patient evaluations of their care and treatment were negatively related with medication errors, i.e., units with patients who were satisfied with their care show less medication errors that units in which patients are not as satisfied with their care.

In summary, the increased share of administrative duties alloted to nurse managers means that they are rarely in the vicinity of patients and nurses. Although nurse managers are responsible for organizing their units, it is equally important that they find sufficient time to support and motivate their staff. However, it is important to note that nurse managers can indirectly improve patient care and outcomes by fostering a safe work environment in their unit.

#### Strengths and limitations

The main limitation of this study was that only three hospitals were involed in the study, from which only 28 units met the inclusion criteria. Accordingly, the study included a small sample of nurse managers because there is usually one (rarely two or more) nurse managers per unit. The small amount of units limited the choice of an appropriate analytic method. Therefore, structural equation modelling was excluded, with analysis of covariance chosen to investigate relationships between the variables [51]. Nevertheless, the fact that 305 nurses and 651 patients participated in the study could be considered a strength when considering that the power analysis specified that 344 nurses and 342 patients should be included to obtain accurate descriptions of the interactions between variables. In addition, we only studied patient satisfaction, nurses’ job satisfaction and medication errors at the unit level.. Hence, the presented results provide information about possible interactions between nurse managers’ work content, nurses’ job satisfaction, patient satisfaction, and medication errors. However, this study could be considered as a pilot study for future outcome studies with larger datasets.

The NMWCQ is a new instrument and, as such, needs to be tested more. It is also important to note that all of the questionnaires (NMWCQ, KUHJSS, and RHCS) are based on self-assessment, which can introduce a certain degree of bias as respondents tend to report overestimates in their evaluations [68]. However, several studies have reported that the KUHJSS and RHCS are reliable and valid instruments. Medication error data from the HaiPro register are based on nurses’ initiative to report medication errors. Therefore, it is impossible to know whether every medication error has been reported. However, it should be noted that HaiPro is the first adverse event reporting system that was introduced in Finland and is now widely used. To gain a representative picture of medication errors, we decided to collect medication error data over 1 year, whereas other data were collected over a time period of approximately 1 month.

Although the study was conducted in Finland, the results can be utilized – to a certain degree - in the evaluation and development of nurse managers’ work on an international level. In the future, it would be interesting to examine whether the hours each registered nurse spent per patient affected patient satisfaction or medication errors. In addition, it would be worthwhile to further develop the NMWCQ and apply it in studies which include far larger samples than what was analyzed in the current study. This means that future studies should involve more hospitals and units than the three investigated in this study. This would allow researchers to use different statistical methods - such as structural equation modeling – to assess the relationships between nurse managers’ work content, nurses’ job satisfaction, patient satisfaction and medication errors. Furthermore, it is important to state that the presented results could be verified by applying different measures of nurse managers’ workload and daily tasks.

## Conclusions

The present study identified several relationships between nurse managers’ work activities, nurses’ job satisfaction, patient satisfaction, and medication errors. In addition, organizational factors - such as the number of nurses per nurse manager and hospital - also influenced nurses’ job satisfaction and medication errors. The findings suggest that nurse managers should focus on improving nursing practices by managing and organizing nurses’ work in a way that makes their employees feel supported, motivated and secure. Furthermore, nurses managers should lead in a way that emphasizes safe and patient-centered care. It would be advisable that the administration at health care organizations critically evaluate nurse managers’ work activities to determine whether the current division of tasks will enable them to meet organizational goals. If not, the organization should proactively develop the work of nurse managers, preferably through collaboration with colleagues, to match what is required in the modern health care organization.

## Supplementary Information

**Additional file 1.** Subscales and items of Nurse Managers Work Content Questionnaire (NMWCQ).

**Additional file 2.** Subscales and items of Kuopio University Job Satisfaction Scale (KUHJSS).

**Additional file 3.** Subscales and items of Revised Humane Caring Scale (RHCS).

## Data Availability

All data supporting our findings were presented within the manuscript.

## Abbreviations

ANCOVA: Analysis of covariance
NMWCQ: Nurse Managers’ Work Content Questionnaire
KUHJSS: Kuopio University Hospital Job Satisfaction Scale
RHCS: Revised Humane Caring Scale
n: number of participants
SD: Standard Deviation
α: Cronbach’s Alpha
*p*: Significance
B: Unstandardized regression coefficient
