# Supplementary material for: Relationships between nurse managers’ work activities, nurses’ job satisfaction, patient satisfaction, and medication errors at the unit level: a correlational study
Source: BMC Health Serv Res. 2021 Apr 1;21:296. doi: 10.1186/s12913-021-06288-5 (PMC8017674; doi:10.1186/s12913-021-06288-5)
Supplement: Supplementary file 1 — Additional file 1. Subscales and items of Nurse Managers Work Content Questionnaire (NMWCQ). [file 12913_2021_6288_MOESM1_ESM.docx]

**Additional file 1. Subscales and items of Nurse Managers Work Content Questionnaire (NMWCQ)**

1. RECRUITMENT
2. I participate in the acquisition of new employees.
3. I look after new employees’ keys, lockers, identity cards, working hours, and IT usernames.
4. I participate in the selection of new employees.
5. I perform/ask for new contracts for new employees.
6. I make decisions regarding leaves of absence.
7. ORGANIZING
8. I make changes to the schedules.
9. I draft the work schedule.
10. I keep the notice board up-to-date (post new notices and remove old ones).
11. I ensure that the schedule is realized.
12. I read, answer and send e-mails.
13. I support and motivate personnel.
14. I check and approve the work schedule.
15. WORK WELLBEING
16. I promote work safety activities for staff in my unit.
17. I promote work protection activities for staff in my unit.
18. I receive and accept sick leave notifications.
19. I have early support conversations with employees.
20. I take part in work health meetings.
21. WORK ATMOSPHERE
22. I manage staff conflicts.
23. I intervene in cases of bad behaviour.
24. I receive and accept temporary care leave (child’s illness) notifications.
25. COMMUNICATION
26. I participate in internet meetings.
27. I make presentations for meetings/training sessions (e.g., creating PowerPoint presentations).
28. I make decisions in situations of educational leave.
29. I prepare meeting minutes, reports, and other releases.
30. I manage unit meetings/personnel conversations/weekly meetings.
31. CLINICAL NURSING
32. I make notes on patients and/or report about patient care.
33. I arrange patient follow-up care.
34. I prepare patients for operations.
35. I participate in unplanned direct nursing.
36. I take care of ordering supplies for the unit (medications, supplies, meals, etc.).
37. I participate in planned direct nursing (e.g., as nurse or charge nurse).
38. I discuss with patients and their relatives.
39. I perform statistics related to patient care (e.g., patient classification).
40. I coordinate beds.
41. DEVELOPMENT OF THE UNIT
42. I organize and promote patient-centred practices and nursing in my unit.
43. I ensure that the organization’s instructions are being followed in the unit.
44. I organize and promote evidence-based practice in my unit.
45. I organize and promote coherent practices in my unit.
46. I prepare and organize how my unit implements the organizational and nursing care strategies.
47. I participate in development projects and studies.
48. I participate in the organization’s internal development (leading work group/ participating in work groups/acting as an expert).
49. I organize the unit’s development activities (development days and projects).
50. I monitor and evaluate nursing outcomes (e.g., pressure ulcers, falls, malnutrition, pain management).
51. I inform and discuss decisions of the hospital and nursing executive with staff.

© Nurmeksela A., Kinnunen J. & Kvist T. 2017

University of Eastern Finland

1. I monitor and evaluate the quality of nursing care (Reporting System for Safety Incidents in Health Care Organizations. announcements, hand hygiene, registration, audits, hospital infections).
2. I discuss nursing guidelines and research information with staff.
3. PERSONNEL DEVELOPMENT
4. I independently take part in additional education (post-graduate studies).
5. I organize student orientation.
6. I evaluate the knowledge of employees.
7. I take care of new employee orientation.
8. I evaluate work performance.
9. I search for and read information related to leadership and management.
10. I search for and read information related to nursing (e.g., evidence-based nursing, clinical practice guidelines, current care guidelines).
11. I complete performance appraisals with employees.
12. DEVELOPMENT OF NURSING
13. I investigate problems and interferences (internal or external) related to nursing processes.
14. I participate in education arranged by employers.
15. I organize orientation and education in my unit (e.g., training on new equipment, information systems, nursing practices).
16. I manage patients’ and relatives’ complaints.
17. FINANCIAL MANAGEMENT
18. I am responsible for my own unit’s purchases (e.g., I act as a contact person for published offers).
19. I handle and check the unit’s invoices.
20. I monitor my own unit’s budget.
21. I publish repair notices regarding broken equipment
22. I participate in my own unit’s budget planning.
23. I meet vendors of medical supplies/equipment.
24. I participate when my own unit plans renovations/new premises.
25. PLANNING AND EVALUATION OF ACTIVITIES
26. I evaluate and plan the actions and nursing processes of the unit.
27. I plan and evaluate how the organization’s strategies (goals) are implemented in my own unit.
28. I plan and evaluate actions and nursing processes with co-operatives.
29. I draft the education plan for the unit.
30. I organize daily activities and ensure work fluency by taking the appropriate action (educations, briefings, presentations, visits, etc.).
31. I draft action plans and reports (along with other reports/ documents).
32. COLLABORATION
33. I collaborate with senior physicians.
34. I collaborate with different professionals (e.g., physicians, special workers, hospital cleaners, multi-professional teams).
35. I collaborate with schools (professional institutes, universities of applied sciences, universities, etc.).
36. I participate in the organization’s shared meetings (e.g., executive teams, clinic meetings).
37. I collaborate with expert nurses regarding the range of responsibility (e.g., student in charge).
38. I collaborate with other organizations (e.g., primary care, private sector., etc.).
39. I collaborate with the charge nurse/team leader.
40. I participate in networking.
41. I collaborate with my professional partner (e.g., assistant nurse manager).
42. I collaborate with the discharge nurse/discharge coordinator.
43. DEVELOPMENT WITH COLLABORATING PARTNERS
44. I collaborate with nurse managers from other units.
45. I collaborate with the nursing director (discussions with own superior, support, motivation).
46. I collaborate with clinical nurse specialists.
47. I collaborate with an education nurse.
48. I participate in nurse managers’ and head nurses’ development days (workgroups/workshops).
49. I collaborate in nurse managers’ common meetings.

© Nurmeksela A., Kinnunen J. & Kvist T. 2017

University of Eastern Finland
