# Supplementary material for: Relationships between nurse managers’ work activities, nurses’ job satisfaction, patient satisfaction, and medication errors at the unit level: a correlational study
Source: BMC Health Serv Res. 2021 Apr 1;21:296. doi: 10.1186/s12913-021-06288-5 (PMC8017674; doi:10.1186/s12913-021-06288-5)
Supplement: Supplementary file 2 — Additional file 2. Subscales and items of Kuopio University Job Satisfaction Scale (KUHJSS). [file 12913_2021_6288_MOESM2_ESM.docx]

**Additional file 2. Subscales and items of Kuopio University Job Satisfaction Scale (KUHJSS)**

(1) Leadership

1. My manager/director is genuinely interested in the well-being of the staff.
2. My manager/director treats the staff fairly and equally.
3. My manager/director encourages staff to take part in the planning of our unit’s operation.
4. My manager/director provides the staff feedback with the aim of developing work.
5. My manager/director informs me thoroughly about issues concerning my unit.
6. My manager/director enables the continuous professional development of the staff.
7. My manager/director is interested in work results and outcomes.

(2) Requiring factors of the work

1. My workload is appropriate.
2. There are usually enough staff in my unit.
3. I do not find my work too stressful.
4. I am satisfied with my working hours.
5. Combining work and personal life is successful.
6. The workload is distributed evenly in my unit.
7. My salary is appropriate in relation to the demands of my work.
8. The upper management of the hospital district appreciates my work.

(3) Motivating factors of the work

1. My work is interesting.
2. I appreciate my own work.
3. I can apply a wide range of my skills and expertise in my work.
4. My work tasks are suitably challenging.
5. Client feedback motivates me in my work.
6. I am willing to work in this hospital district in the future.

(4) Working environment

1. My unit has appropriate work facilities.
2. My unit has appropriate equipment to ensure quality of care.
3. My work unit is comfortable.
4. My work unit is safe and secure.

(5) Working welfare

1. I look after my personal well-being.
2. I am happy with my current health.
3. I am active in developing myself professionally.
4. I feel I am a competent employee.

(6) Participation in decision-making

1. I have opportunities to make independent decisions in my work.
2. I have opportunities to plan my work independently.
3. I have a chance to influence decision-making in my unit.
4. I have a chance of career development in the hospital district.

(7) Sense of community

1. I trust the expertise of my colleagues.
2. There is a good community spirit in my unit.
3. The flow of information works well in my unit.
4. New employees are welcomed in my unit

© Kvist et al. 2012

University of Eastern Finland
