# Supplementary material for: Relationships between nurse managers’ work activities, nurses’ job satisfaction, patient satisfaction, and medication errors at the unit level: a correlational study
Source: BMC Health Serv Res. 2021 Apr 1;21:296. doi: 10.1186/s12913-021-06288-5 (PMC8017674; doi:10.1186/s12913-021-06288-5)
Supplement: Supplementary file 3 — Additional file 3. Subscales and items of Revised Humane Caring Scale (RHCS). [file 12913_2021_6288_MOESM3_ESM.docx]

**Additional file 3. Subscales and items of Revised Humane Caring Scale (RHCS)**

1. Professional practice
2. I was appreciated
3. I was accepted for what I was
4. I was listened to when I had worries
5. The staff showed just the right level of interest
6. The staff relied on my own assessment of how I felt
7. I was able to discuss issues with the staff in confidence
8. My treatment was based on my needs
9. I felt welcomed into the hospital
10. I felt safe in hospital
11. Sufficient concern was shown about my state of health
12. I received help when I needed it
13. I was treated with respect
14. I was treated in a friendly way
15. My pain was noticed and taken seriously
16. The atmosphere was positive
17. The nursing staff were professional
18. The physicians were professional
19. Information and participation in own care
20. I was able to speak with the staff in private
21. I received enough information about my illness
22. Restrictions relating to my illness were explained to me in a way that I could understand
23. I received enough information about my medication
24. I was able to ask questions concerning my care
25. The rules and restrictions relating to the hospital environment and the ward community were explained to me in a way that I could understand
26. I received enough information about my home care
27. I was given clear instructions about home care
28. I was addressed in clear and intelligible language
29. I was able to participate in the planning of my care
30. My family were given enough attention
31. Cognition of physical needs
32. I was given enough to drink
33. I was given an appropriate amount of food
34. I was helped with my personal hygiene if necessary
35. I was able to maintain and/or improve my mobility
36. Human resources
37. The staff had enough time for me
38. There were enough members of staff
39. The atmosphere was unhurried

5) Pain and apprehension

1. I received medication for my pain at the right time
2. As well as using medicine, my pain was relieved with other treatments (e.g. changing my position, physical care)
3. I was given guidance about pain treatment in a way that I could understand
4. My fears were alleviated
5. Interdisciplinary collaboration
6. There was good collaboration between members of staff
7. The other staff were professional
8. The members of staff respected each other’s expertise
9. Outcome variables
10. I set clear goal for my care together with the staff
11. The goal of my care was received
12. I am satisfied with the outcome of my care
13. I am satisfied with my care

© Kvist et al. 2013

University of Eastern Finland
